# Treatment in a Geriatric Day Hospital improve individualized outcome measures using Goal Attainment Scaling

**DOI:** 10.1186/s12877-016-0397-9

**Published:** 2017-01-07

**Authors:** Paige Moorhouse, Olga Theou, Sherri Fay, Miranda McMillan, Heather Moffatt, Kenneth Rockwood

**Affiliations:** 1 Division of Geriatric Medicine, Dalhousie University, Veterans’ Memorial Building, 5955 Veterans memorial Lane, Halifax, Nova Scotia B3H2E1 Canada; 2 Centre for Health Care of the Elderly, Nova Scotia Health Authority, Veterans’ Memorial Building, 5955 Veterans memorial Lane, Halifax, Nova Scotia B3H2E1 Canada

**Keywords:** Geriatric day hospital, Goal attainment scaling, Mobility

## Abstract

**Background:**

Evidence regarding outcomes in the Geriatric Day Hospital (GDH) model of care has been largely inconclusive, possibly due to measurement issues. This prospective cohort study aims to determine whether treatment in a GDH could improve individualized outcome measures using goal attainment scaling (GAS) and whether improvements are maintained 6-months post-discharge.

**Methods:**

A total of 469 outpatients admitted to a Canadian Geriatric Day Hospital, between December 2008 and June 2011, were included in the analysis (81.1 ± 6.7 years, 66.3% females); a smaller cohort of 121 patients received a follow-up phone call 6 months following discharge. Baseline, discharge and 6 month post-discharge observer-rated measures of mobility, cognition, and function were completed using GAS. Traditional psychometric measures were also captured.

**Results:**

The mean number of goals set was 1.6 (SD 0.8) and patients set goals in the following domains: 88% mobility or falls reduction; 18% optimization of home supports; 17% medication optimization;12% cognition; 8% increasing social engagement; and 5% optimization of function. Total GAS was the most responsive measure to change with 86% of patients improving at discharge; mobility goals were the most likely to be achieved. Six-month GAS scores remained significantly higher than GAS scores on admission. Those who had more goals were more likely to improve during GDH admission (OR 1.49, CI 1.02-2.19) but this association was not seen 6 months after discharge.

**Conclusions:**

This study demonstrated short- and long-term effectiveness of GDH in helping patients achieve individualized outcome measures using GAS.

## Background

Geriatric Day Hospitals (GDH) were developed in the United Kingdom in the late 1950s to help bridge the gap between inpatient and community care for older adults [1]. They aim to foster functional independence (rehabilitative and medical care), and reduce the risk of more serious conditions (preventative care) for community-dwelling older adults through comprehensive assessment and management [2]. GDH teams usually consist of a physician, nurse, social worker, physiotherapist, occupational therapist, psychologist, and dietician. Despite evidence that selected multidisciplinary interventions may improve outcomes in some participants, such as decreased risk of falls, the medical literature regarding the long-term outcomes of the day hospital model of care delivery has been largely inconclusive [3–6] in part due to heterogeneity of services provided and outcome measures used [7, 8].

The challenge of which tools should be used to demonstrate meaningful outcomes in day hospital patients is well-recognized [7]. Many traditional, standardized psychometric measures have shown responsiveness in day hospital populations; [5, 9] however, these measures tend to de-emphasize clinical judgment in favour of maximizing reliability, which may limit validity [8]. Their limitations include: choice of instrument complicating comparisons of effectiveness between programs; responses not being analyzed for subgroups of admitted patients; and limiting responsiveness to a specific domain.

In older patients with multiple health issues, individualized outcome measures, such as Goal Attainment Scaling (GAS) may provide a more relevant, scalable, and patient-centered alternative [8]. In contrast to psychometric tests, in which the referent is the population average performance, GAS is a clinometric score in that the referent is the individual. GAS allows for measurement of an unlimited breadth and number of clinically important outcomes, and has demonstrated validity, feasibility, reliability and responsiveness for community dwelling older adults including those undergoing rehabilitation [8, 10, 11]. Also GAS has been endorsed as a measure that can improve patient-centredness by focusing care on what patients want and judging performance, at least in part, by how patients’ goals are met [12, 13].

Despite its clinical usefulness, data on GAS in day hospital populations is currently limited to one study [3] which showed that 39% of patients deteriorated after discharge from hospital. Even so, this study did not include patients with known dementia (an increasingly prevalent condition) and did not provide subgroup analyses to investigate what factors could affect changes [3]. We set out to determine whether treatment in a GDH could improve individualized outcome measures using GAS and whether these improvements were maintained 6-months post-discharge. Also we investigated whether achievement of the goals of the patients who attend the GDH was a function of the goals themselves, the patient’s frailty, or other factors.

## Methods

### Setting and participants

This prospective cohort study took place in the GDH at the Nova Scotia Health Authority (Central Zone) in Halifax, Canada. The GDH follows the traditional day hospital model of care and assesses over 200 patients annually. The average length of enrollment is 11 visits (Canadian national average 18) [7] with typically 2 visits/week. The program runs daily from Monday to Thursday with four patient cohorts attending Monday and Wednesday (mornings or afternoons), or Tuesday and Thursday (mornings or afternoons). Daily census rates average 20 patients. The program is staffed by two separate but overlapping teams each consisting of a physician, a registered nurse, a physical therapist, an occupational therapist and a social worker. Baseline comprehensive multidisciplinary assessments examine cognition, mobility, function, co-morbidities, symptom control and polypharmacy. In addition observer-rated GAS is completed as part of routine care by GDH team members for all patients at admission and discharge.

**Fig. 1:**
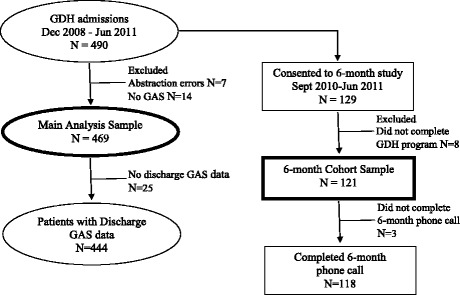
Patient Flowchart

A total of 490 outpatients were serially admitted to the GDH between December 2008 and June 2011 (Fig. 1). The data of 21 patients were excluded from this study due to abstraction errors (*N* = 7) or missing admission GAS scores (*N* = 14). Of the remaining 469 patients, 25 had no discharge GAS data. Starting September 2010 all admitted patients were asked to receive a follow-up phone call 6 months following discharge (*n* = 206). Consent was obtained from 129 patients (henceforth referred to as the 6 month cohort). Among the 77 who did not consent, 24 declined participation, 14 withdrew from GDH program before consenting, 10 were admitted to hospital or other service before consenting, seven did not end up attending the GDH program, three did not provide a reason, 14 were excluded for other reasons, and for five we missed the opportunity to obtain consent.

Among the 129 who consented, eight did not complete the GDH program and three did not participate in the 6-month follow up phone call leaving a total of 118 patients included in the 6-months follow up analyses (Fig. 1). Follow up GAS data were obtained by a research nurse using a standardized and validated telephone interview [3] with either the patient or their caregiver (in cases where the patient was deceased or cognitive impairment was noted during GDH admission). The 6-month follow-up period was selected with the objective of allowing enough time to lapse to show change, but not so much time that new health issues may confound the score [3]. Ethics approval for the study was obtained from the Capital Health Research Ethics Board.

### Goal Attainment Scaling (GAS)

GAS is an individualized, observer-rated, outcome measure designed to capture patient-centred treatment effects on a five-point scale [8]. At admission, problem areas specific to each patient were identified by the GDH multidisciplinary team and set as goals. The team aims to assign goals for the patients that are the most pertinent to the patient (i.e. reason for referral or of concern to the patient), measurable and realistic to achieve during the GDH. Typically 1–3 goals are assigned per patient; for additional areas of concern for the patients, the team does not assign goal levels to these domains but monitors them. The GDH team groups the goals into four general domains (mobility, function, cognition, other). For goals in the “other” domain, the research team reviewed all goals and grouped them by consensus as social, home support, and medication. Therefore for purposes of analyses, the goals set at admission were grouped into six domains including mobility, cognition, falls, medication optimization, home supports and social issues.

The admission status for each goal was captured descriptively and set at “0” on the scale. Plausible outcome levels representing degrees of improvement or worsening were documented and designated to one of the four remaining levels of the scale: (i.e., −2 = very much worse than baseline; −1 = somewhat worse; +1 = somewhat better; and +2 = very much better). For example, the GDH team set a mobility goal for patient Y, who reported having one fall per week and difficulty with stairs (admission status = 0). The desired outcomes for this patient were identified as having less than one fall/week and following the recommendations of the GDH team regarding stairs (+1 “somewhat better”) and having no further falls and no difficulty with stairs (+2 “much better”). At GDH discharge the goals were reviewed by the GDH team and the level of goal attainment (i.e., the score) was determined by the extent to which the current status conformed to one of the levels defined at admission (no evident change from baseline was scored as 0). During the 6 month follow up telephone interview, the research nurse enquired about the current level of functioning for each original goal and then scored goal attainment in accordance with the scale defined at admission. The research nurse was blinded to the goal attainment scores assigned at discharge.

GAS scores are standardized by a summary formula that adjusts for varying numbers of goals per patient and varying levels of attainment per goal:

$$ \mathrm{GAS} = 50+\left\{\left(10*\sum {\mathrm{w}}_{\mathrm{i}}{\mathrm{x}}_{\mathrm{i}}\right)/\left({\left(0.7*\sum {\mathrm{w}}_{\mathrm{i}}+0.3*{\left(\sum {\mathrm{w}}_{\mathrm{i}}\right)}^2\right)}^{1/2}\right)\right\} $$

where x_i_ is the individual attainment level (between −2 and +2) and w_i_ is the weight of the goal (in this study all goals were weighted as 1). The formula results in a score of 50 when all goals remain at 0 (as is the case at admission when goals are first set), <50 when there is net worsening across goals, and >50 when there is net improvement across goals. An overall GAS score (Total GAS) was calculated for each patient, as well as scores for goals by domain.

### Other health measures

Patients attending the GDH routinely complete a battery of standardized, validated psychometric measures upon admission and discharge (Table 1). The Mini-Mental State Examination (MMSE) [14] is a brief, 20-item instrument that measures orientation to time and place, immediate recall, short-term memory, calculation, language and constructive ability. The maximum obtainable score is 30 and the published minimum detectable change (MDC) is 3 points [15, 16]. The BERG Balance Scale (BBS) [17] is a 14-item functional measure of balance impairment in ambulatory older individuals, with an MDC of 5–7 points depending on baseline [18]. The Performance Oriented Mobility Assessment (POMA) [19] is a widely-used assessment of mobility, balance and gait in older populations. The maximum score is 28 (12 points for the gait component and 16 points for the balance component) and the MDC is 5 points [20]. The Timed Up and Go (TUG) [21] is an evaluation of transfers and ambulation with an MDC of 4 seconds [22, 23]. The Elderly Mobility Scale (EMS) [23] assesses mobility in 7 dimensions of functional performance including locomotion, balance and key position changes for a total score of 20. Its MDC is 3 points [24]. The Lawton Brody Instrumental Activities of Daily Living scale [25] is a validated assessment of function in older adults with an MDC of 1 point [26].

**Table 1:** Outcome measures and their minimum detectable change

Measure	Description	On admission	On discharge	Minimum detectable change
The Mini Mental Status Examination (MMSE)	30-point test of cognition	✔	✔	3 points [15, 16]
Berg Balance Scale (BBS)	14-item functional measure of balance impairment	✔	✔	5-7 points depending on baseline [17, 18]
The Performance Oriented Mobility Assessment (POMA)	Assessment of mobility, balance and gait: 12 points for the gait component and 16 points for the balance component	✔	✔	5 points [19, 20]
The Timed Up and Go (TUG)	Evaluation of sequential locomotor tasks including chair transfers and ambulation with well-established norms	✔	✔	4 seconds [21–23]
Elderly Mobility Scale (EMS)	20-point multidimensional assessment of mobility in frail elderly patients	✔	✔	3 points [23, 24]
Lawton Brody	Validated scale to measure instrumental activities of daily living	✔	✔	1 point [25, 26]
Frailty Index	37 variables describing the proportion of accumulated deficits in an individual (baseline frailty)	✔		N/A
Goal Attainment Scaling (GAS)	Clinometric scale describing changes in patient directed goals	✔	✔	Any change

Frailty at admission was operationalized using the deficit accumulation approach. A frailty index was constructed following a standard methodology [27] by combining 37 measures collected as a part of the standard assessment upon admission to the GDH [(e.g. weight loss, mobility impairment, and osteoporosis). Each of the included variables was coded to represent the presence or the absence of a health problem. A frailty index score, ranging from 0 to 1, was calculated for each patient by dividing the number of health problems the patient had by 37; if a patient had X missing data then the denominator was adjusted (i.e., 37-X). The characteristics of the frailty index constructed for this study were similar to those from other clinical databases. The frailty index is designed to be used as a continuous measure, however, cut points have been suggested to identify frailty groups: 0 to ≤ .10 non frail, >.10 to ≤ .21 vulnerable, >.21 to <0.45, mildly/moderately frail, ≥0.45 severely frail [28].

### Statistical Analyses

Statistical analyses were carried out using IBM SPSS Statistics (version 22) and R Studio (version 0.98.1103). Descriptive statistics and comparisons between groups (e.g., those in the main cohort who were followed up at 6-months vs. those who were not and those who improved on GAS vs. those who did not) were carried out with t-tests and Chi squared analyses. Changes in psychometric measures (admission to discharge) and GAS (admission, discharge, 6 months follow up) were examined using repeated measures ANOVA. The responsiveness of each measure was compared by calculating the standardized response mean (mean change divided by the standard deviation of the change scores) [29] and the proportion of patients who improved by at least the established MDC values (note that for GAS any improvement is considered clinical significant and was recorded as MDC). We performed univariate logistic regression analyses and one way ANOVAs to determine the relationship between baseline characteristics and improvement on Total GAS at discharge and 6 months.

## Results

The main cohort included 469 sequential patients (34% male) with a mean age of 81.1 ± 6.7 years (range 62–99) and a mean length of enrollment of 58 ± 19 days (Table 2). Patients were referred by general practitioners (47%), geriatricians (33%), other specialists (10%), emergency department (6%) and community sources (4%). Reasons for referral included falls (59%), mobility impairment (31%), Parkinson’s disease (3%), cognitive impairment (2%), medication/polypharmacy (2%), and other reasons (3%). At baseline, participants had mild impairment of cognition, moderate to severe impairment of mobility, and impairment of Instrumental Activities of Daily Living (Table 2). Among the 446 participants with valid frailty scores, 3% were non frail, 22% vulnerable, 65% mildly/moderately frail, and 10% severely frail. The mean number of goals set per patient was 1.6 ± 0.8. Goals were most commonly set for mobility or falls reduction (88%), followed by optimization of home supports (18%), medication optimization (17%), cognition (12%), increasing social engagement (8%), and optimization of function (5%).

**Table 2:** Baseline characteristics for the main cohort and the 6-month follow-up cohort by GAS-improvement status at discharge and 6 months, respectively

Baseline Characteristic mean (SD) unless otherwise indicated	Main cohort			6-months follow up cohort		
	All	Improvement on GAS at discharge	No improvement on GAS at discharge	All	Improvement on GAS at 6 months	No improvement on GAS at 6 months
*N*	469	381	63	121	99	19
Age	81.1 (6.7)	81.2 (6.8)	80.7 (6.6)	80.8 (6.7)	80.9 (6.7)	79.8 (7.0)
Sex [female n (%)]	311 (66.3%)	251 (65.9%)	40 (63.5%)	84 (69.4%)	70 (70.7%)	12 (63.2%)
Program length (days)	58.1 (19.0)	59 (17.6)	54.1 (25.2)	60.8 (23.8)	61.5 (25.8)	55.5 (10.8)
MMSE	25.6 (3.8)	25.7 (3.7)	25.4 (3.8)	26.1 (3.3)	26.1 (3.4)	25.5 (2.5)
BBS	37.3 (9.7)	37.7 (9.3)	36.1 (10.9)	38.2 (9.5)	37.8 (9.8)	39.3 (7.9)
POMA	9.6 (3.3)	9.7 (3.3)	9.3 (3.6)	9.9 (3.6)	9.7 (3.3)	10.1 (3.3)
EMS	16.1 (2.9)	16.2 (2.8)	15.9 (3.3)	16.5 (2.8)	16.4 (2.9)	16.5 (2.6)
Lawton Brody	3.7 (2.1)	3.5 (2.0)	4.0 (2.4)	3.0 (1.9)	3.0 (1.9)	3.0 (1.7)
TUG	22.8 (11.9)	22.4 (10.5)	24.7 (18.5)	22.2 (10.5)	22.5 (10.5)	21.4 (10.9)
Frailty Index	0.30 (0.11)	0.29 (0.11)	0.31 (0.12)	0.26 (0.10)	0.26 (0.10)	0.27 (0.11)
GAS, number of goals	1.64 (0.81)	1.67 (0.83)	1.44 (0.76)*	1.52 (0.76)	1.55 (0.80)	1.37 (0.60)

*Significantly different than those who improved at discharge (*p* = 0.04)

*BBS* BERG Balance Scale; *EMS* Elderly Mobility Scale; *GAS* Goal Attainment Scaling; *MMSE*, Mini-Mental State Examination; *POMA*, Performance Oriented Mobility Assessment; *TUG* Timed Up and Go

Comparison of discharge scores to admission scores revealed that all measures improved statistically except for the MMSE (*p* = 0.09). Total GAS was the most responsive measure to change with 86% of patients improving at discharge (Standardized Response Mean 1.62) (Table 3). Only four patients declined in Total GAS at discharge and due to this small number, when examining changes in GAS, these patients were combined with those who remained stable (*N* = 59). Looking at goals by domain, more patients improved on mobility and home support goals (Table 3).

**Table 3:** Outcomes, change and responsiveness by health measure between GDH admission and discharge for main cohort

Measure	*N*	Admission mean (SD)	Discharge mean (SD)	Change mean (SD)	Standardized Response Mean	% Achieving Positive MDC
MMSE	371	25.6 (3.6)	25.9 (4.2)	0.32 (3.66)	0.09	22%
BBS	399	37.7 (9.5)	44.3 (8.1)*	6.63 (6.32)	1.05	61%
POMA	397	9.7 (3.3)	12.0 (2.8)*	2.34 (2.75)	0.85	20%
EMS	405	16.2 (2.9)	17.9 (2.9)*	1.68 (3.08)	0.55	57%
Lawton Brody	396	3.6 (2.0)	3.7 (2.0)*	0.12 (0.75)	0.16	6%
TUG	398	22.4 (10.8)	18.1 (8.0)*	−4.33 (7.49)	−0.58	48%
GAS Total	444	50	62.6 (7.8)*	12.56 (7.76)	1.62	86%
GAS Mobility	412	50	61.9 (8.0)*	11.93 (8.03)	1.49	82%
GAS Cognition	56	50	57.9 (7.3)*	7.90 (7.33)	1.08	64%
GAS Function	23	50	58.3 (5.8)*	8.26 (5.76)	1.43	74%
GAS Medication	78	50	62.8 (9.4)*	12.80 (9.38)	1.36	79%
GAS Home Supports	85	50	58.9 (5.2)*	8.86 (5.21)	1.70	80%
GAS Social	35	50	57.7 (4.3)*	7.71 (4.26)	1.81	77%

*Significantly different from admission (*p* < 0.05)

*BBS* BERG Balance Scale; *EMS* Elderly Mobility Scale; *GAS*, Goal Attainment Scaling; MDC- minimum detectable change; *MMSE*, Mini-Mental State Examination; *POMA*, Performance Oriented Mobility Assessment; *TUG* Timed Up and Go

The 121 patients who were followed up 6 months after discharge had lower (*p* < 0.001) frailty index (0.26 ± 0.10 vs. 0.31 ± 0.12) and Lawton Brody scores (3 ± 1.9 vs. 3.9 ± 2.1) at baseline compared to the 348 patients who were not followed up; all other baseline characteristics were similar. At discharge those who were followed up 6 months had lower Lawton Brody scores (3.1 ± 1.9 vs. 3.9 ± 2.0) and higher GAS scores (Total 65.3 ± 7.2 vs. 61.5 ± 7.7; mobility 64.4 ± 7.2 vs. 61.0 ± 8.1; cognition 65 ± 9.3 vs. 56.7 ± 6.3; function 62.9 ± 4.9 vs. 56.3 ± 5; medication 68.2 ± 11.2 vs. 61.4 ± 8.4); all other discharge characteristics were similar. Among the 6-month cohort, improvement at 6-months compared to admission remained evident for Total GAS and all GAS domains except cognition and function; the sample size for those domains was less than 10 patients (Table 4).

**Table 4:** GAS change from admission at discharge and 6 months following discharge for the 6-months follow up cohort

Measure	*N*	Discharge mean (SD)	6-months mean (SD)	% of patients who improved	
				Discharge	6 months
				N (%)	N (%)
GAS Total	118	65.3 (7.3)*	61.4 (8.1)*#	113 (96%)	99 (84%)
GAS Mobility	109	64.4 (7.1)*	61.1 (8.1)*#	103 (94%)	90 (83%)
GAS Cognition	8	65.0 (9.3)*	53.8 (9.2)	7 (88%)	5 (63%)
GAS Function	6	63.3 (5.2)*	56.7 (10.3)	6 (100%)	4 (67%)
GAS Medication	15	68.7 (11.3)*	58.3 (6.4)*#	13 (87%)	11 (73%)
GAS Home Supports	17	60.0 (3.5)*	58.8 (6.0)*	16 (94%)	13 (76%)
GAS Social	8	58.8 (3.5)*	58.8 (6.4)*	7 (88%)	6 (75%)

*Significantly different from admission (*p* < 0.05)

#Significantly different from discharge (*p* < 0.05)

GAS, Goal Attainment Scaling

Using improvement as the outcome, in univariate logistic regression analyses, those who had more goals, were more likely to improve during GDH admission (OR for number of goals 1.49, CI 1.02-2.19, *p* = 0.04) but these associations were not seen at 6 months after discharge. None of baseline characteristics (i.e., age, sex, frailty index, length of enrollment, and baseline cognitive, functional or mobility psychometric scores) predicted improvement during GDH stay or at 6 months after discharge (*p* > 0.05). Findings were similar when comparing the characteristics of those patients who experienced improvement during GDH stay to those who did not improve during GDH stay (Table 2).

## Discussion

The GDH model provides a solution to locally and nationally identified programmatic needs. On a national level, the importance of outpatient rehabilitative programs for reducing costs and improving outcomes is well recognized [30]. Previous studies examining outcomes in GDH have relied upon numerous psychometric measures [5] or GAS alone [3]. GAS appears to be a responsive measure to change in this population. Our results are consistent with and build upon those of Crilly et al. [3] as this GDH population is larger and includes those with dementia. This study also appears to demonstrate maintenance of benefit from GDH at 6 months post discharge. This is in contrast to the study of Malone et al. [5], which found that gains in mobility or function were not maintained at 3 months in a population with baseline characteristics similar to the current study. It is difficult to know whether the responsiveness of GAS explains the difference in longer term outcomes between these two studies.

Although responsive, GAS cannot be readily used as a means to select optimal candidate patients for the GDH service since none of the patient baseline characteristics predicted response to rehabilitation. Therefore no single baseline measure appears to predict overall GDH performance [31] or longer term maintenance of improvements. While this finding may seem discouraging from a patient selection perspective, it also suggests that adaptive interventions such as the GDH, can effectively serve a wide range of people. Although not an optimal selection tool, the use of GAS could streamline patient assessment on admission and discharge by reducing the number of psychometric measures that are completed for each patient. One of the current criticisms of the use of multiple psychometric measures in rehabilitative programs is that they are time consuming [30]. Second, a streamlined approach to outcome measurement within the GDH may allow front line care workers to spend less time charting outcomes, and more time in direct patient care.

Our study has several important limitations. The patients who were followed up 6 months after discharge had lower frailty and higher functional ability at baseline and higher function and GAS scores at discharge compared to those patients who were not followed up. No comparison groups were added and no measures other than GAS were repeated at 6 months. Also GAS scores at 6 months after discharge were based on patient or (in the case of cognitively impaired patients) caregiver report (vs. GDH care team observations at admission and discharge). On the other hand, patient-centred interventions need to produce changes that patients or caregivers can on average recognize as meaningful [13].

## Conclusions

This study demonstrated short- and long-term effectiveness of GDH in helping patients achieve individualized outcome measures using GAS. The GDH therefore has the potential to help answer the call of our growing population of frail older adults who typically benefit more from a continuum of community care than from the traditional admit-discharge model of our health care system [3, 7]. Future studies dealing with the cost effectiveness of GDH interventions may benefit from using a GAS outcome measure in order to understand the economic case as it relates to individualized outcomes that matter to patients.

## Abbreviations

BBS: BERG Balance Scale
CI: Confidence Interval
EMS: Elderly Mobility Scale
GAS: Goal attainment scaling
GDH: Geriatric Day Hospital
MDC: Minimum detectable change
MMSE: Mini-Mental State Examination
OR: Odds ratio
POMA: Performance Oriented Mobility Assessment
SD: Standard deviation
TUG: Timed Up and Go
